# Supplementary material for: Delicate Hybrid Laponite–Cyclic Poly(ethylene glycol) Nanoparticles as a Potential Drug Delivery System
Source: Pharmaceutics. 2023 Jul 21;15(7):1998. doi: 10.3390/pharmaceutics15071998 (PMC10384068; doi:10.3390/pharmaceutics15071998)
Supplement: Supplementary file 1 [file pharmaceutics-15-01998-s001.zip › pharmaceutics-2338896-supplementary.pdf]

Supplementary Materials

1. UV-Vis determination of DOX loading efficiency

2.0 mg of DOX·HCl was dissolved in 0.4 ml of water to make an aqueous DOX solution (5 mg/mL). Meanwhile, 8 mg of LAP-cPEG or LAP-mPEG was dissolved in 8 mL of Di-water. Then, 0.16 mL of the DOX aqueous solution was added to 8 mL of LAP-cPEG, and LAP-mPEG solution, respectively. The mixtures were stirred for 24 h at room temperature in the dark. The resulting solutions were dialyzed against water (150 mL) in the dark using a membrane with a MWCO of 15,000. The dialysis media were replaced and collected every 9 h. After 4 times of dialysis cycles, the combined dialysis media (600 mL) were concentrated by rotatory evaporation in reduced pressure to 79.18 mL and 118.0 mL, respectively, with an UV absorbance (480 nm) of 0.06469 and 0.06558, correspondingly. A standard DOX calibration curve (480 nm) was made based on series of DOX standard solutions at 0, 0.0001,0.0005, 0.00625, 0.0125 and 0.05 mg/mL (Figure S1).

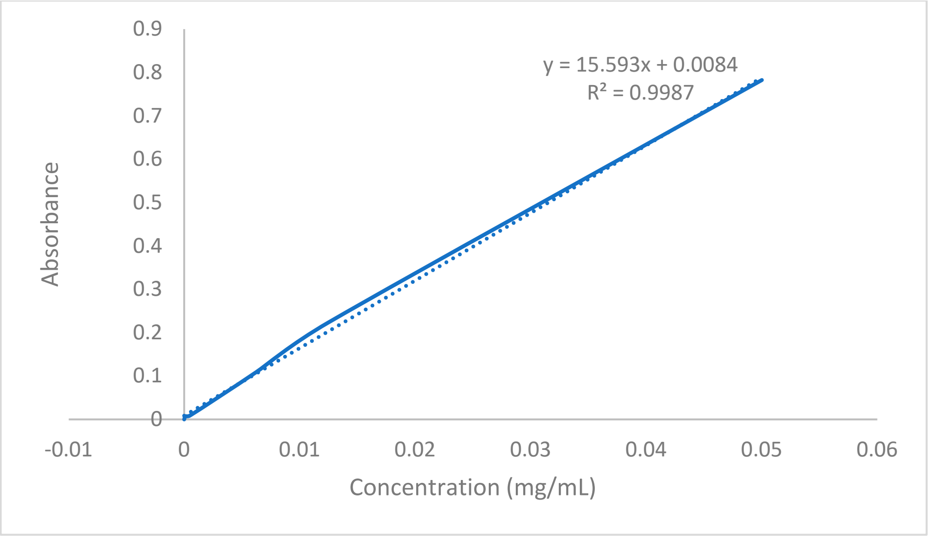

Figure S1. Calibration curve of DOX in water at 480 nm

The corresponding concentrations of DOX in concentrated dialysis media were obtained based the regression equation; thus, the DOX loading efficiencies were determined using the following equation:

DOX loading efficiency = (Mass of feeding DOX - Mass free DOX)/ (Mass of feeding DOX) ×100% as listed in Table S1.

Table S1. The summary of calculation of the DOX loading efficiency

Nanosystem	Absorbance	[DOX] (mg/mL)	Free DOX (mg)	Encapsulated DOX (mg)	Efficiency (%)
LAP-cPEG	0.06469	0.003610	0.2858	0.5142	64
LAP-mPEG	0.06558	0.003667	0.4327	0.3673	46

*Free DOX (mg) = [DOX]× Volume of concentrated dialysis media

** Feeding DOX = 0.16 mL×5 mg/mL=0.8 mg

On the other hand, the LAP-cPEG/DOX and LAP-mPEG/DOX formulation solutions inside the dialysis membrane bags collected were 8.65 mL and 8.70 mL, respectively. Thus, the LAP-cPEG/DOX and LAP-mPEG/DOX formulations corresponded a DOX concentration at 0.059mg/mL and 0.042 mg/mL, respectively.

2. In vitro DOX release profile

DOX release profiles were monitored by measuring an increase in UV absorbance. Before applying the formulation for release study, the LAP-cPEG/DOX solution was concentrated to a half volume, increasing DOX concentration to 0.118 mg/ml from 0.059mg/mL. To 1.8 mL of the LAP-cPEG/DOX formulation solution was added 0.2 mL of PBS or human plasma. The mixture was transferred to a dialysis bag with a MWCO of 15,000 and immersed in 18 mL of PBS while stirring. At predetermined time points, t=0, 0.33, 1, 3, 6, 12, 24, 48 h, 0.6 mL of external PBS buffer was withdrawn for UV absorbance tested at 480 nm. An equal volume of fresh PBS (0.6 mL) was replenished in the dialysis medium. For LAP-mPEG/DOX release study, the LAP-mPEG/DOX solution was similarly concentrated to one-third volume, increasing DOX concentration to 0.126 mg/ml from 0.042 mg/mL. 1.8 mL of the LAP-mPEG/DOX formulation was mixed with 0.2 mL of plasma and processed the release study. 0.2 mL of plasma mixed with 1.8 mL of LAP-cPEG (2mg/mL) or LAP-mPEG (3mg/mL) solution was conducted as a control experiment. The absorbances at different time points were listed in Table S2.

Table S2. The absorbances of withdrawn dialysis medium at different time points

Time (h)	0	0.33	1	3	6	12	24	48
LAP-cPEG/DOX-PBS	0.000025	0.01047	0.01422	0.03719	0.03434	0.03471	0.03778	0.037[illegible]
*LAP-cPEG/DOX-Plasma	-0.003475	0.01276	0.02384	0.03581	0.05140	0.1269	0.1518	0.062[illegible]
**LAP-mPEG/DOX-Plasma	-0.000771	0.008786	0.02465	0.05614	0.1654	0.1476	0.09423	0.078[illegible]

*Subtracting "LAP-cPEG in plasma" from "LAP-cPEG/DOX in plasma" at same time point

** Subtracting "LAP-mPEG in plasma" from "LAP-mPEG/DOX in plasma" at same time point

The corresponding DOX concentrations [DOX] in PBS medium at time points t=0, 0.33, 1, 3, 6, 12, 24, 48 h, were determined (Table S3) using standard DOX calibration curve (Figure S1).

Table S3. The concentrations of DOX (mg/mL) in dialysis medium at different time points

Table S3. The concentrations of DOX (mg/mL) in dialysis medium at different time points

Time (h)	0	0.33	1	3	6	12	24	48
LAP-cPEG/DOX-PBS	-0.000537	0.0001331	0.0003732	0.001846	0.001664	0.001687	0.001884	0.001859
LAP-cPEG/DOX-Plasma	-0.000762	0.0002797	0.0009902	0.001758	0.002758	0.007603	0.009196	0.003486
LAP-mPEG/DOX-Plasma	-0.000588	0.0000248	0.001042	0.003062	0.01007	0.008927	0.005504	0.004465

The released DOX percentages from the LAP-cPEG/DOX formulation at different time points were calculated using eqs: DOX release (%) =(20×[DOX])/ (1.8×0.118) × 100.

The released DOX percentages from the LAP-mPEG/DOX formulation at different time points were calculated using eqs: DOX release (%) =(20×[DOX])/ (1.8×0.126) × 100. The final calculated results were listed in Table S4 and present as Figure 8a.

Table S4. DOX release percentages from the formulations at different time points

Time (h)	0	0.33	1	3	6	12	24	48
LAP-cPEG/DOX-PBS	-5.05	1.25	3.51	17.4	15.7	15.9	17.7	17.5
LAP-cPEG/DOX-Plasma	-7.17	2.63	9.32	16.6	26.0	71.6	86.6	32.8
LAP-mPEG/DOX-Plasma	-5.19	0.22	9.19	27.0	88.8	78.7	48.5	39.4

2. Integrations of representative corresponding areas for LAP-cPEG

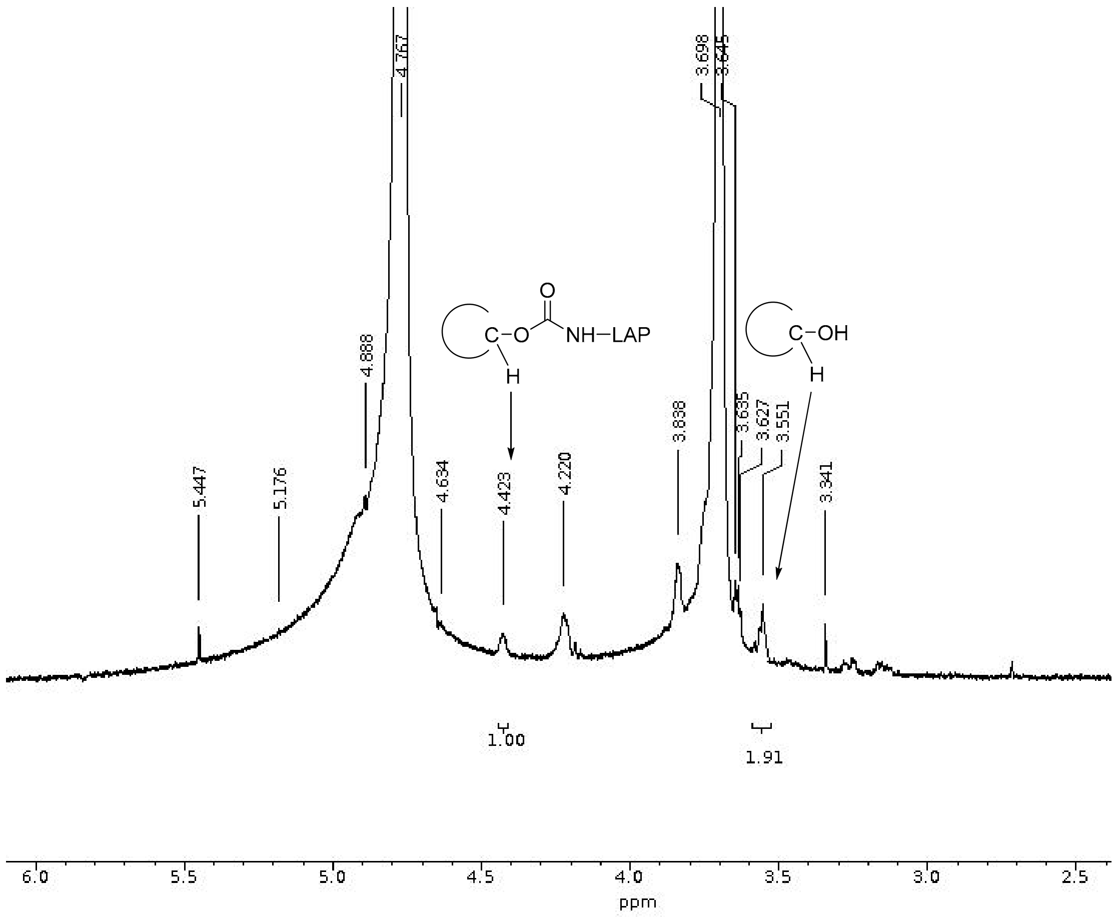

Figure S2. Representative peak integration values of LAP-cPEG system.

3. FTIR and NMR analysis of LAP-cPEG or LAP-mPEG nanoparticles

For FTIR test, solid LAP, LAP-NH_2, LAP-cPEG or LAP-mPEG was directly applied on the sample clamp of the FTIR device; for NMR test, LAP, LAP-NH_2, LAP-cPEG or LAP-mPEG was spread in deuterium oxide (D_2O) to make a 2 mg/mL solution.

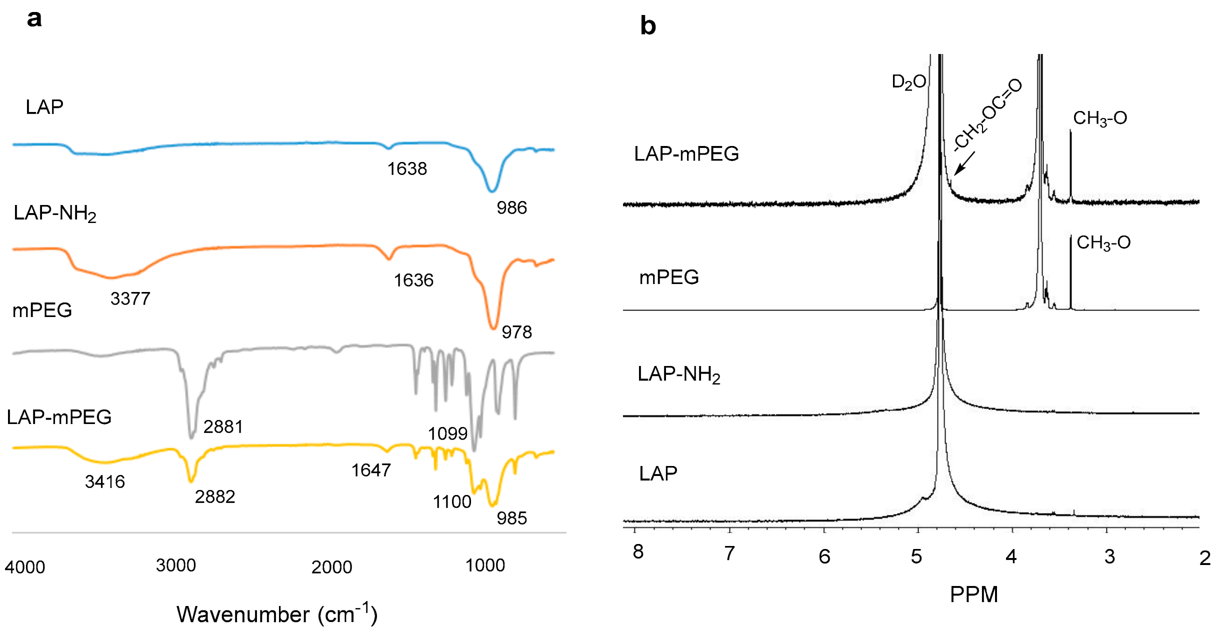

Figure S3. FTIR spectra (a), and ^{1}H NMR spectra (b) of LAP, LAP-NH_2, mPEG and LAP-mPEG.

4. Visible experiments of LAP, LAP-NH_2, LAP-mPEG, and LAP-cPEG solutions

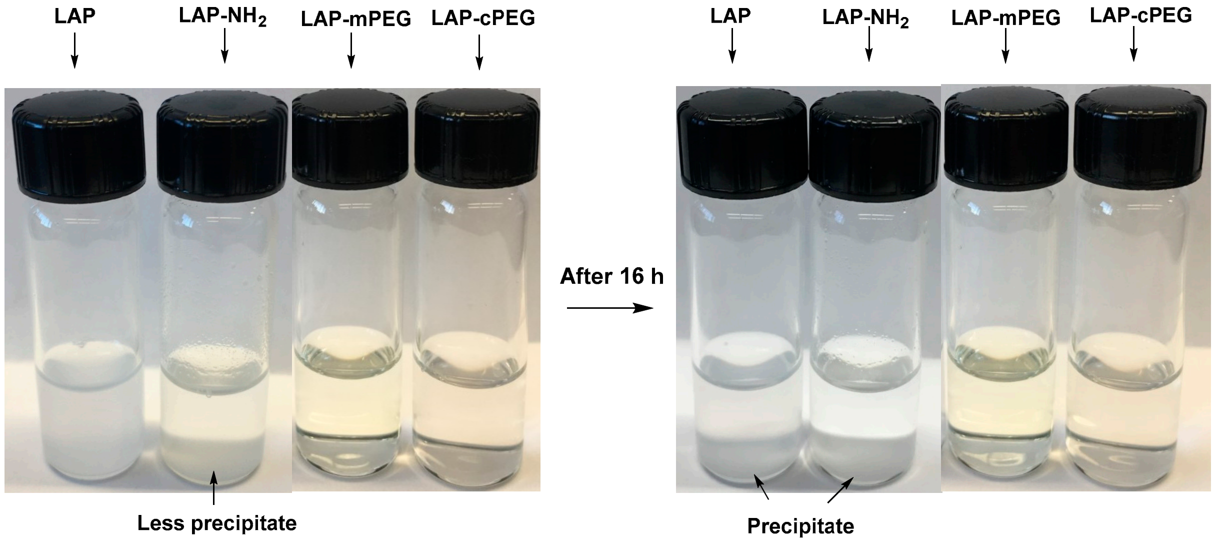

Figure S4. LAP and LAP-NH_2 suspensions (1 mg/mL) Vs LAP-mPEG and LAP-cPEG solutions (5 mg/mL).

5. Remove free DOX from LAP/DOX, LAP-mPEG/DOX, or LAP-cPEG/DOX by dialysis with 15K membrane

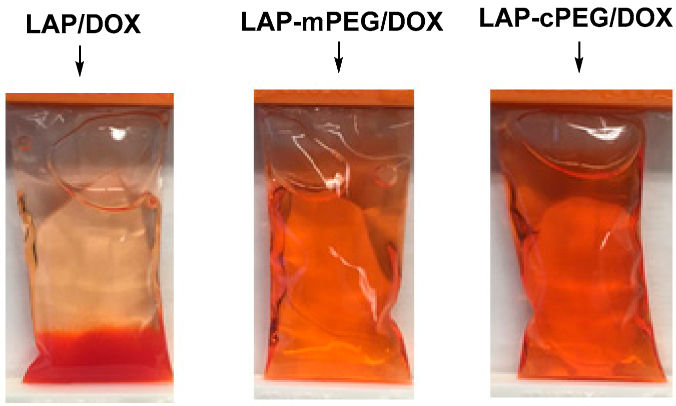

Figure S5. LAP/DOX, LAP-mPEG/DOX, and LAP-cPEG/DOX formulations are subjected in 15 K dialysis membranes after 6 h.

6. Ultra performance liquid chromatography (UPLC)

With high solubility and stability of LAP-cPEG/DOX formulation, UPLC was used to characterize the complex. It was performed on a Waters Acquity System using a photodiode array detector (detection at 485 nm). DOX solution was freshly prepared by dissolving Doxorubicin hydrochloride in deionized water with a concentration of 0.025 mg/mL while the LAP-cPEG/DOX formulation was two-fold diluted with deionized water. Two sample solutions (3 µL) were injected into a BEH C18 column (100 × 2.1 mm, 1.7 µm), and elution was performed at a flow rate of 0.2 mL/min with a linear gradient mode using two mobile solvents, eluent A (0.1% TFA/water (v/v)) and B (0.1% TFA/acetonitrile (v/v)). The sample elution began with a mobile phase 1% B (0–2.0 min) which was followed by a linear increase to 80% B (13.4 min), a decrease to 50% B (13.8 min), a decrease to 1% B (14.4 min) and finally an isocratic elution at 1% B (18 min).

7. Cytotoxicity of LAP, LAP-mPEG and LAP-cPEG

A549 Cells were seeded into 96-well plates (15,000 cells/well) and incubated overnight. Concentrated solution of LAP, LAP-mPEG or LAP-cPEG was added to reach the concentrations of 0.01, 0.1, 0.5 and 1.0 mg/mL and incubated for 48 h. After this time, the medium was removed, cells were washed with fresh medium. PBS and XTT reagents were then added, and the plate was again incubated for 1 h. With the Synergy HT plate reader (BioTek Instruments; Winooski, VT) the absorbances (OD) could be measured as an indication of cell viability, using the optical density differences at 690 nm and 492 nm.

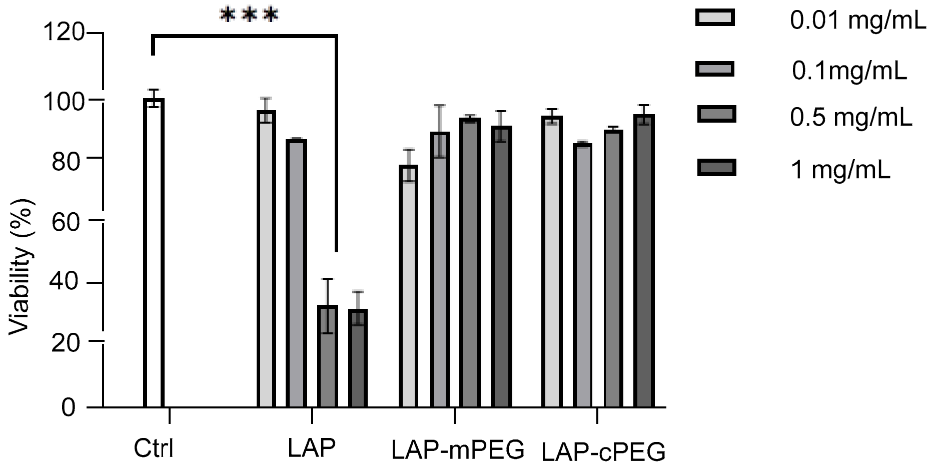

Figure S6. In vitro cell cytotoxicity test of raw LAP, LAP-mPEG, and LAP-cPEG by means of XTT assay. The control indicated the cell growth medium only. With high concentration (e.g., 0.5 mg/mL), raw LAP showed significant cytotoxicity, while LAP-PEG exhibited no toxicity even at very high concentration (e.g., 1 mg/mL) (***$P < 0.001$ for significance).

8. Copies of NMR (500 MHz) spectral Data

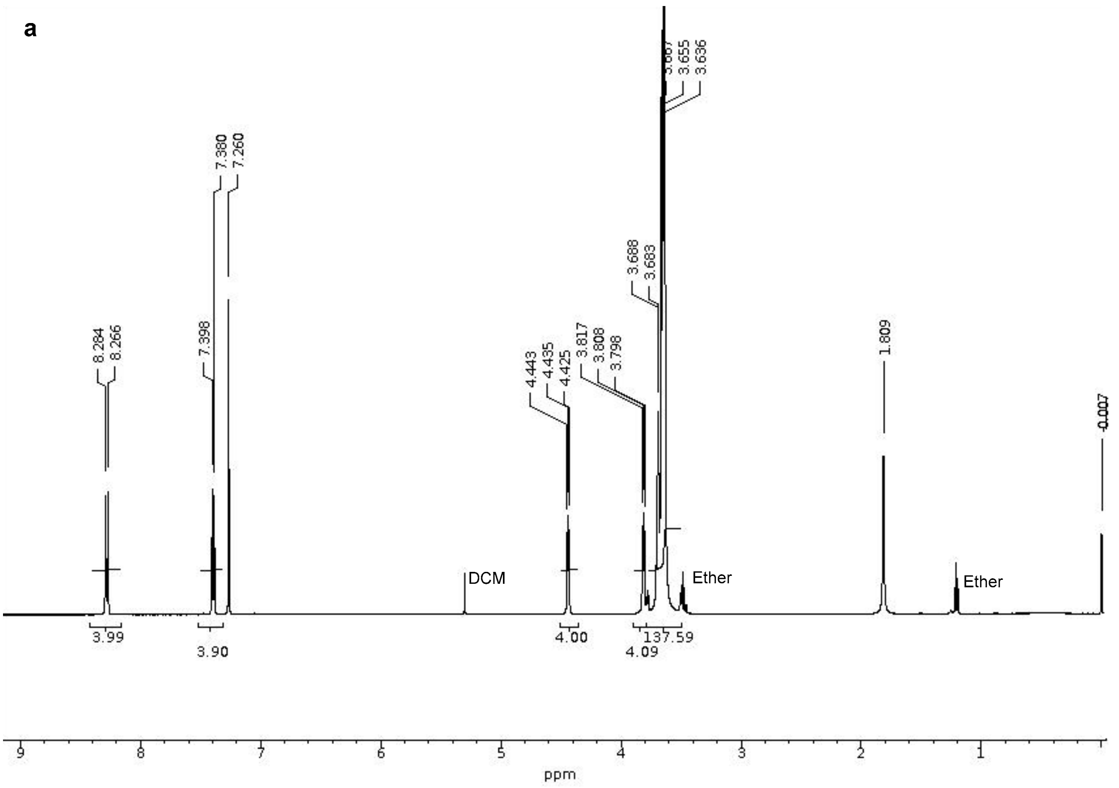

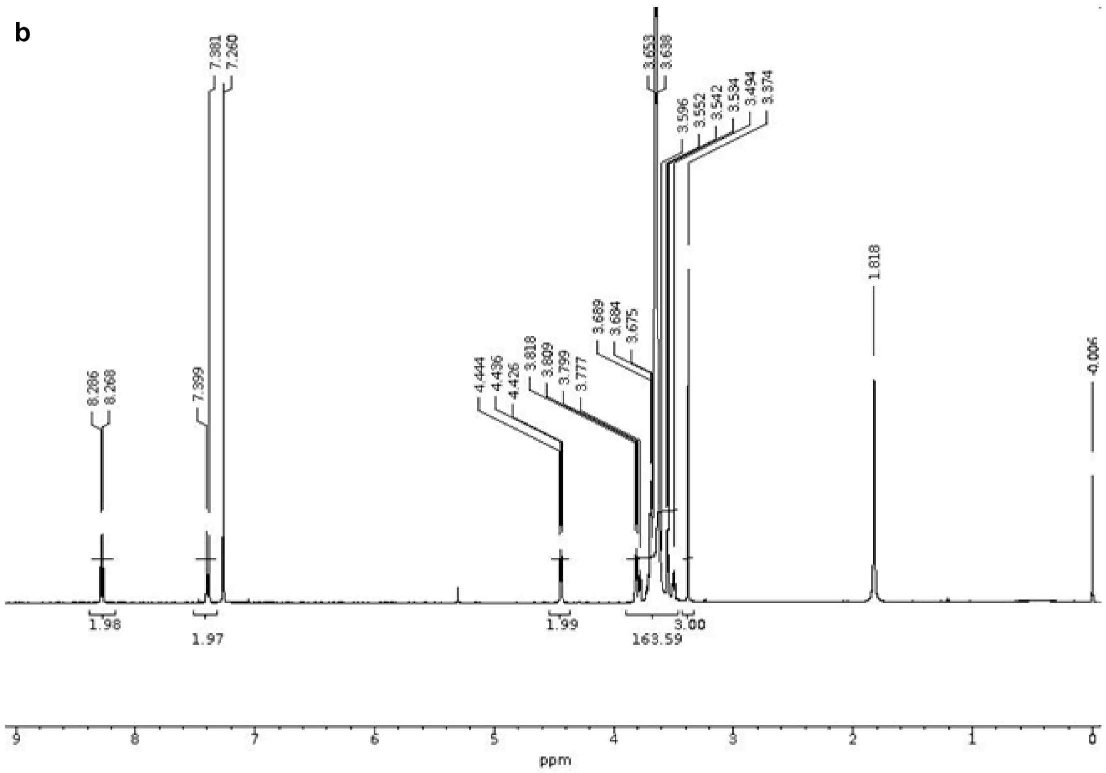

Figure S7. ^{1}H NMR spectrum of PEG-NP (a) and mPEG-NP (b) in $CDCl_3$.

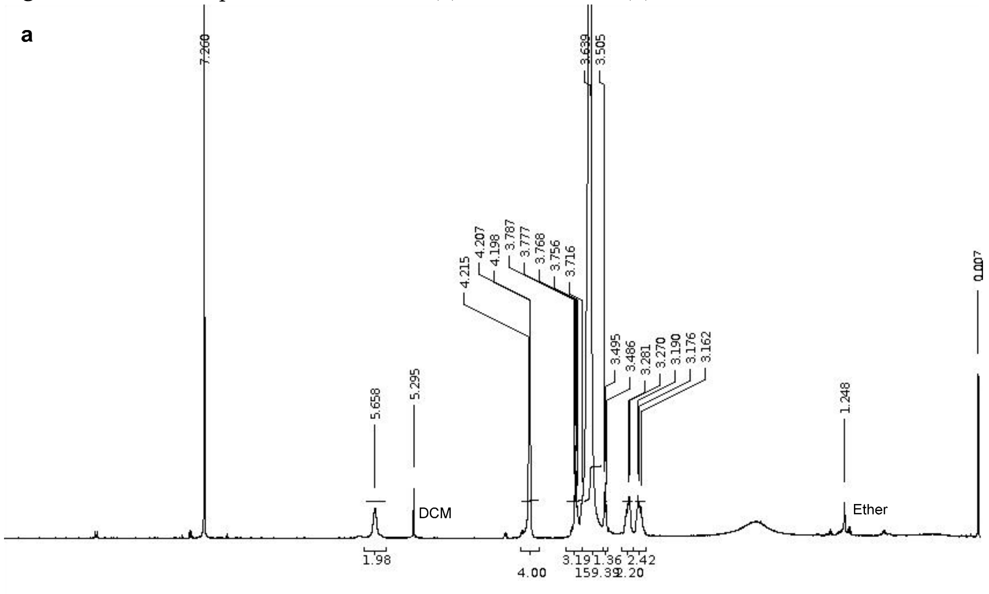

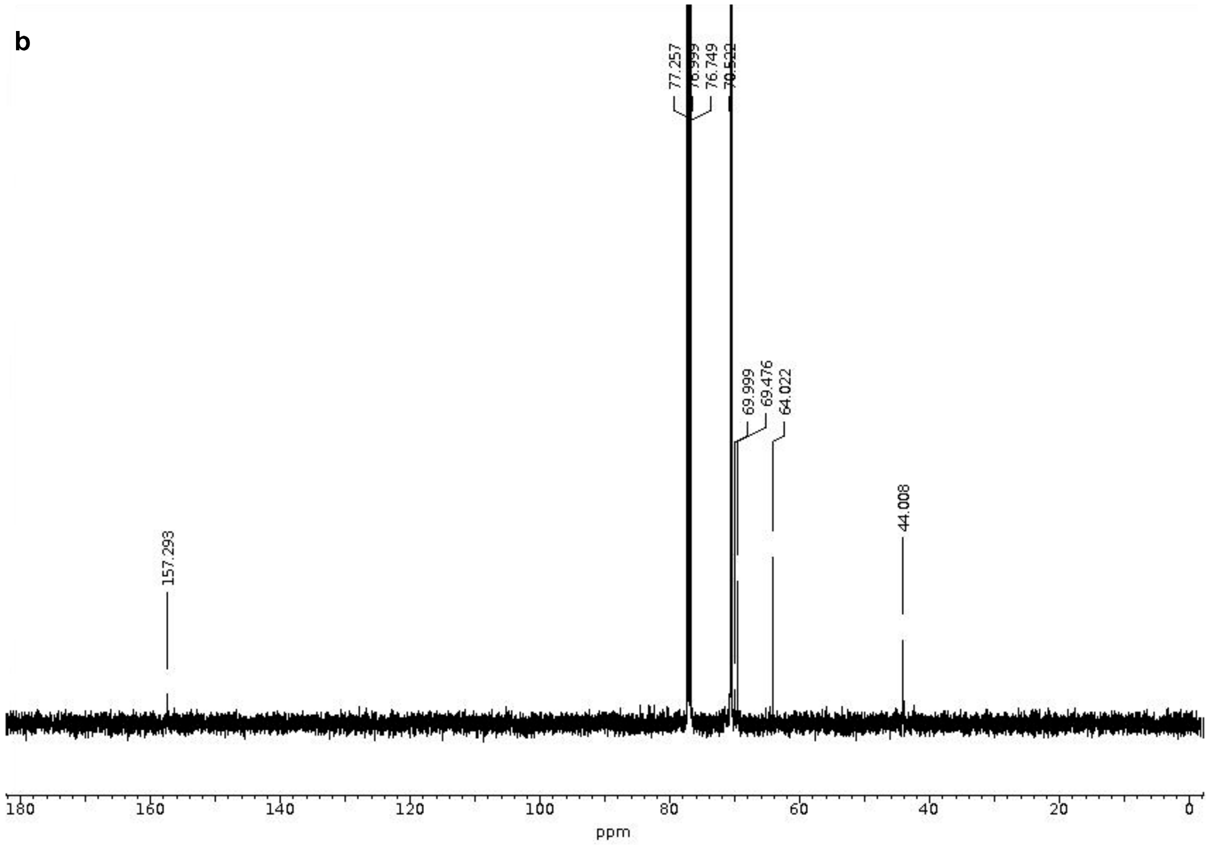

Figure S8. ^{1}H NMR spectrum (a) and ^{13}C NMR spectrum (b) of cPEG-OH in $CDCl_3$.

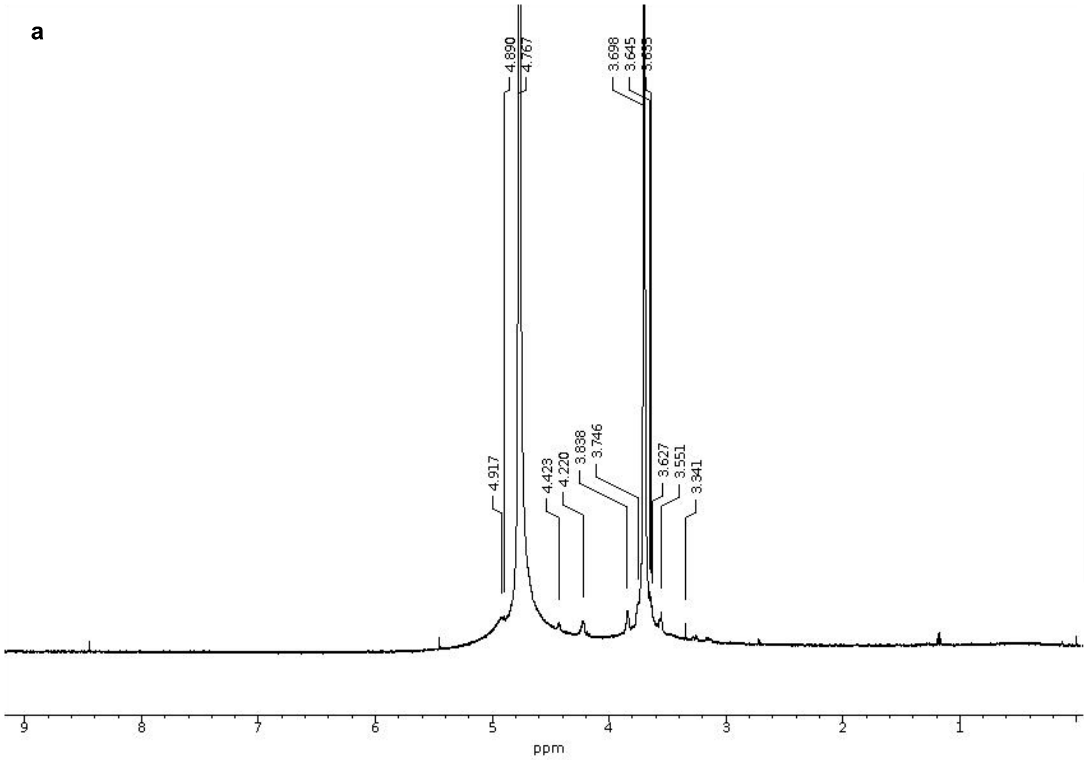

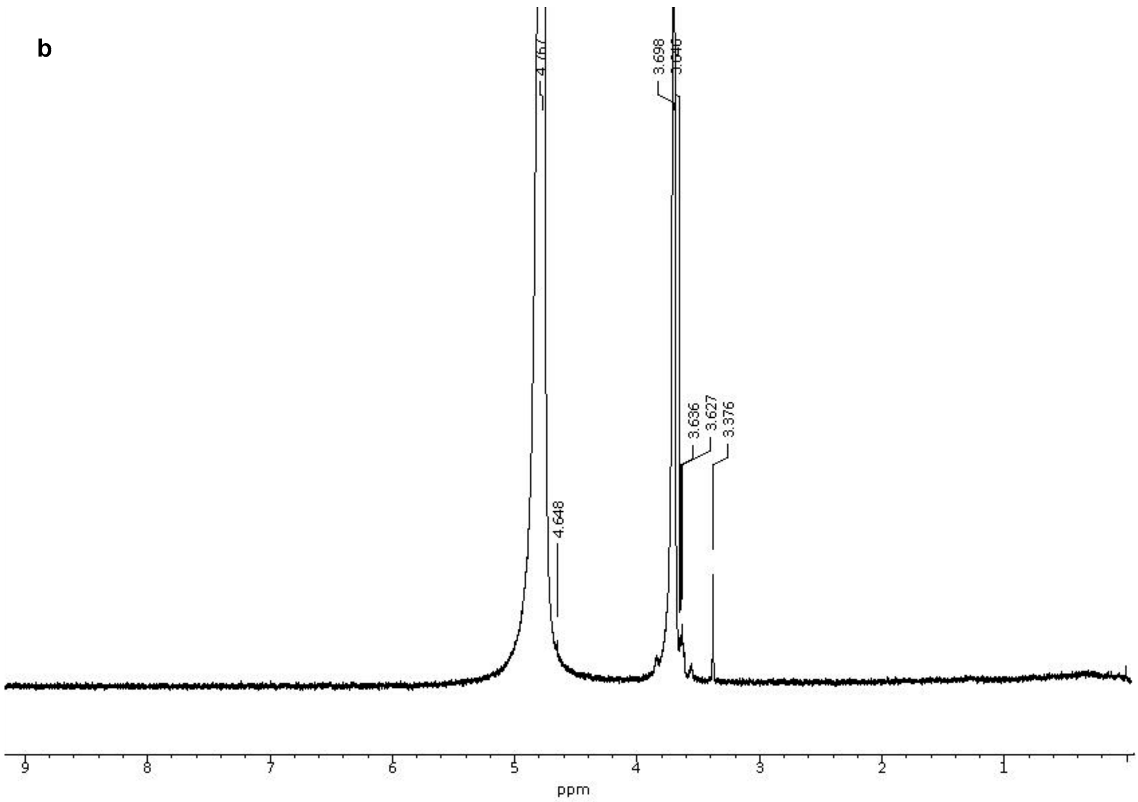

Figure S9. ^{1}H NMR spectrum of LAP-cPEG (a), and LAP-mPEG (b) in D_2O.
